# Effects of different combined regimens of cisplatin, metformin, and quercetin on nasopharyngeal carcinoma cells and subcutaneous xenografts

**DOI:** 10.1038/s41598-020-80198-0

**Published:** 2021-01-13

**Authors:** Zhongwei Chen, Zhen Zeng, Shanshan Zhu, Ying Zeng, Qihuang Lin, Lianzhong Luo, Xuan Hong

**Affiliations:** 1 Xiamen Key Laboratory of Marine Medicinal Natural Products Resources, Xiamen Medical College, Xiamen, 361023 People’s Republic of China; 2 Fujian Province Universities and Colleges Engineering Research Center for Marine Biomedical Resources Utilization, Xiamen Medical College, Xiamen, 361023 People’s Republic of China

**Keywords:** Phenotypic screening, Chemotherapy

## Abstract

Cisplatin, metformin, and quercetin are all reliable anticancer drugs. However, it is unclear how effective their different combination regimens are on the growth of nasopharyngeal carcinoma cell line Sune-1 and subcutaneous xenograft in nude mice. This study evaluated the effects of single-drug, two-drug, and three-drug simultaneous or sequential combined application of these drugs on the growth of Sune-1 cells and subcutaneous xenograft tumors in nude mice. The results showed that the different combination regimens of cisplatin, metformin and quercetin all had significant inhibitory effects on the proliferation of Sune-1 cells and the growth of subcutaneous xenografts in nude mice (*P* < 0.01), and the inhibition rate of the three drugs simultaneous combined application was significant Higher than the two-drug combination or single-drug application (*P* < 0.05), the contribution level of each drug in the three-drug combination application from high to low were cisplatin > metformin > quercetin. In summary, our results indicate that the simultaneous combination of cisplatin, metformin, and quercetin may synergistically inhibit the growth of Sune-1 cells and subcutaneous xenografts in nude mice through their different anticancer mechanisms, which may be clinically refractory and provide reference for chemotherapy of patients with recurrent nasopharyngeal carcinoma.

## Introduction

Nasopharyngeal carcinoma (NPC) is a unique type of head and neck carcinoma, which is common in Southeast Asia and southern China, where it brings significant economic pressure and health burden^1^. In routine clinical therapy, radiotherapy is the most commonly used method, followed by chemotherapy. Cisplatin is a commonly used prototype platinum-based chemotherapeutic drug that plays an important role in the therapy of carcinoma, it has been used for 50 years so far^2,3^. However, in the procedure of chemotherapy, cisplatin therapy has serious poison and side effects such as ototoxicity, nephrotoxicity, cardiotoxicity and neurotoxicity, etc., and its clinical application is greatly restricted^4^. Therefore, the exploitation and utilization of new chemotherapeutic drugs and the research and application of new combined chemotherapy regimens, thereby reducing the poison and side effects of chemotherapeutic drugs and improving the therapeutic effect of patients with nasopharyngeal carcinoma are urgent problems to be solved.

Metformin (1,1-Metformin Hydrochloride, Met) is considered an insulin sensitizer and can reduce insulin resistance by restoring insulin sensitivity^5^. However, some studies have shown that metformin also has anti-tumor effects in vivo and in vitro. Saeki et al. found that metformin can achieve anti-tumor effects by activating AMPK and inhibiting mTOR^6^. Pabona et al. found that metformin acts directly in endometrial cancer cells, which can prevent endometrial cancer by modifying the expression and signal network of sterol receptors^7^. In addition, some studies have found that metformin combined with cisplatin can increase the inhibitory effect on cancer. For example, Lee et al. found that metformin can enhance the therapeutic effect of cisplatin on triple-negative breast cancer through the expression of RAD51^8^. Moro et al. found that metformin and cisplatin can synergistically resist KRAS/LKB1 co-mutant tumors, and may prevent or delay the onset of cisplatin resistance by targeting CD_133_^+^ cancer stem cells^9^. Quercetin belongs to flavonoids and is widely distributed in a variety of vegetables such as onions, ginger, celery, etc., fruits such as apples, strawberries, etc., and Chinese herbal medicines such as ginkgo, notoginseng, sophora, etc^10^. Some studies have proved that quercetin has significant anti-tumor activity, which can inhibit cancer cell proliferation, induce cancer cell apoptosis, interfere with cancer cell cycle and signal transduction pathways, and reverse cancer cell multidrug resistance^11^. Similarly, some studies have shown that the combined use of quercetin and cisplatin can also significantly increase the inhibitory effect on cancer. For example, Xin Li and other studies have found that the combined use of quercetin and cisplatin can inhibit the phosphorylation of Akt and IKKβ, and cause Inhibition of NF-κB and anti-apoptotic protein xIAP, thereby inhibiting the growth of oral squamous cell carcinoma in mice^12^. Zhao et al. found that quercetin has inhibitory activity on hepatocellular carcinoma cells through p16-mediated cell cycle arrest and apoptosis, and its combination with cisplatin has a synergistic inhibitory effect in inhibiting cell growth and inducing apoptosis^13^. However, whether the combined chemotherapy of metformin, quercetin and cisplatin can improve the efficacy of nasopharyngeal carcinoma has not been discussed. Therefore, we used the Cell Counting Kit-8 (CCK-8) to evaluate the effects of different combined regimens of cisplatin, metformin, and quercetin on the proliferation of Sune-1 cells, as well as the effects on human nasopharyngeal carcinoma subcutaneous xenografts, to provide a theoretical reference for the chemotherapy of patients with clinically refractory and recurrent nasopharyngeal carcinoma.

## Methods

### Cell culture

Human nasopharyngeal carcinoma cell line Sune-1 was purchased from ATCC(American type culture collection). Sune-1 cells were cultured in DMEM medium (ThermoFisher, USA) containing 10% fetal bovine serum (ThermoFisher, USA), 100 U/mL penicillin and 100 U/mL streptomycin. Cells were maintained in a humid atmosphere with 5% CO_2_ at 37 °C.

### Drug treatment

Cisplatin, quercetin and metformin were purchased from Sigma Biological Company (USA). Based on the reported plasma peak concentration (PPC) and pre-experiment results, the concentration gradient of cisplatin, metformin, and quercetin was determined as follows: cisplatin^14^ was 0, 0.25, 0.5, 1, 2, 4, 6, 8, 10, 12 μg/mL, metformin^15^ was 0, 0.25, 0.5, 1, 2, 4, 6, 8, 10, 12 mmol/L, and quercetin^16^ was 0, 10, 20, 30, 40, 50, 60, 70, 80, 90 μmol/L. When the drugs was applicated in combination, the low, medium and high concentrations were set according to the IC_50_ values of cisplatin, metformin and quercetin, which were expressed as: CDDP-L, CDDP-M, CDDP-H, Met-L, Met M, Met-H, Qu-L, Qu-M, Qu-H.

### CCK-8 assay

Sune-1 cells were cultured in EMDM medium containing 10% FBS, and digested with 0.25% trypsin to make a cell suspension of 1 × 10^5^ cells/mL. 200 μL of cell suspension per well was inoculated into 96-well plates, and cultured in an incubator at 37 °C, 5% CO_2_ saturation humidity for 24 h, then a series of concentrations of cisplatin, metformin and quercetin were added to the 96-well plates to determine the cell proliferation. The experiment was divided into a blank group, a control group and a drug treatment group. The drug treatment group was further divided into: (1) single-drug group: low-concentration, medium-concentration and high-concentration of cisplatin, metformin and quercetin were added separately; (2) The two-drug combination group, including simultaneous combined application and sequential combined application, the optimal medication order of the two-drug combination was determined first, and then the optimal administration concentration was determined, the different combination regimens are shown in Fig. 2 and Table 2; (3) The three-drug combination group was divided into simultaneous combined application and sequential combined application. The different combinations are shown in Fig. 3.

100 μL of each concentration of the drug was added in the single-drug group. In the two-drug combination group, after concentrating the two drugs twice, add 50 μL each to keep the drug concentration and volume in each well consistent. And in the three-drug combination group, after concentrating the drugs 4 times, add 25 μL each, and then add 25 μL mediums to keep the drug concentration and volume consistent. After being cultured in a 37 °C 5% CO_2_ incubator for 48 h, add 10 μL of CCK-8 reagent to each well and continue culturing for 4 h. The optical density value of each well at a wavelength of 450 nm was determined by a fully automatic microplate reader (ThermoFisher, USA ). The cell suppression rate was calculated, the formula^17^ is: cell inhibition rate = [(control group-drug treatment group)/(control group-blank group)] × 100%.

### Evaluation of combined application of two drugs

Fractional product concept (fp value)^18^ was used to evaluate the property of the interaction between the two drugs, fp value = I_1+2_/(I_1_ + I_2 _− I_1_ × I_2_), where I_1+2_: the average cell inhibition rate of the combined effect of the two drugs, I_1_ and I_2_: The average inhibition rate of the drug acting alone. If the fp value is greater than 1, the action of the two drugs is synergistic; if the fp value is 1, the action of the two drugs is additive; if the fp value is less than 1, the action of the two drugs is antagonistic.

### L16 (4^3^) orthogonal experiment

Based on the IC_50_ values of cisplatin, metformin, and quercetin, set 4 levels of 0, low, medium, and high concentrations, respectively, and select the orthogonal array of L16(4^3^) according to the number and level of parameters, as shown in Table 3. The concentration of cisplatin varied from 0 to 8.6 μg/mL, metformin varied from 0 to 8.7 mmol/L, and quercetin varied from 0 to 73 μmol/L. According to the combined form in the orthogonal array, the combination of three drugs was separately prepared to further act on Sune-1 cells, and CCK-8 was used to determine the cell inhibition rate. In order to analyze the optimized combination of three drugs and the contribution level of the three drugs.

### Construction of subcutaneous xenograft tumor model

SPF grade 8-week-old male BALB/c nude mice, weighing 16–18 g, were purchased from Xiamen University Animal Experiment Center (License number: SYXK(min)2018-0009), and were raised under the condition of no specific pathogens in the Xiamen University Animal Experiment Center. All animals were kept in a pathogen-free environment and fed ad lib. The procedures for care and use of animals were approved by the Ethics Committee of the Xiamen University and all applicable institutional and governmental regulations concerning the ethical use of animals were followed. These animals were allowed to adapt to the living conditions for 5 days before the experiment, then Sune-1 cells in the logarithmic growth phase were made into a cell suspension of 5 × 10^6^ cells/mL and injected subcutaneously into nude mice.

### Animal grouping and intervention

After the xenograft tumor grew, 30 nude mice were randomly divided into 5 groups for drug intervention. The grouping was as follows: A: control group, intraperitoneal injection of normal saline every 12 h; B: cisplatin group, intraperitoneal injection of cisplatin (0.1 g/mL) followed by injection of normal saline 12 h later; C: quercetin group, Intraperitoneal injection of quercetin (2.5 g/mL) followed by injection of normal saline 12 h later; D: Cisplatin → metformin group, intraperitoneal injection of cisplatin (0.1 g/mL) followed by metformin (1.5 g/mL) 12 h; E: quercetin + cisplatin + metformin group, intraperitoneal injection of a mixture of quercetin (2.5 g/mL), cisplatin (0.1 g/mL) and metformin (1.5 g/mL), injection of normal saline 12 h. Each group was administered once a day, 100 μL/nude mouse, for 5–8 days. During the experiment, the maximum diameter (LD) and minimum diameter (SD) of the tumor were measured every 3 days, and the calculation formula^19^ of tumor volume (V) = LD × SD^2^ × 0.52, tumor growth rate, and tumor growth inhibition rate (TGI) were calculated. Compare and analyze the difference of single-drug, two-drug combination and three-drug combination. Calculation formula of tumor growth rate = tumor volume in treatment group / tumor volume in the control group × 100%, tumor growth inhibition rate calculation formula = 1-tumor growth rate^20^.

### Statistical analysis

SPSS 13.0 was used for statistical processing, pairwise comparison was performed by t test analysis, and comparison between groups was by one-way ANOVA. *P* < 0.05 was considered statistically significant.

## Results

### Anti-proliferation effect of single drug on Sune-1 cells

**Figure 1:**
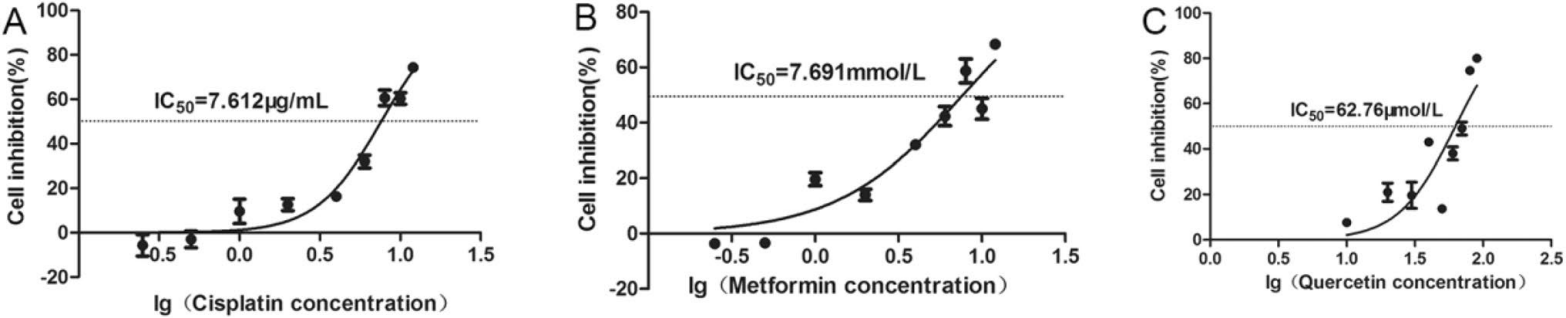
lg drug concentration-cell inhibition rate curve of different drugs on Sune-1 cells. (**A**) cisplatin, (**B)** metformin, (**C)** quercetin.

The proliferation inhibitory effect of different concentrations of cisplatin, metformin, and quercetin on Sune-1 cell line was determined by CCK-8 experiment. The results showed that the inhibitory effects of cisplatin, metformin, quercetin on Sune-1 cells increased with increasing concentration. Figure 1 shows the IC_50_ values of these three drugs on Sune-1 are 7.612 μg/mL, 7.691 mmol/L, and 62.76 μmol/L, among which cisplatin has the lowest IC_50_ value on Sune-1 cells, indicating that Sune-1 cells are sensitive to cisplatin, the degree is stronger than the other two drugs, followed by quercetin.

According to the IC_50_ values of cisplatin, metformin, and quercetin, set the low, medium, high concentration, respectively as follows: CDDP-L, CDDP-M, and CDDP-H are 3.3, 7.6, and 8.6 μg/mL; Met-L, Met-M, Met-H are 3.4, 7.7, and 8.7 mmol/L; Qu-L and Qu-M, Qu-H are 53, 63, and 73 μmol/L, respectively. Furthermore, the effect of a single drug on Sune-1 was determined, the results showed that, compared with the control group, cisplatin, metformin, and quercetin alone had a significant inhibition on Sune-1 cells (*P* < 0.01). And the higher concentration of the drug, the higher inhibitory effect of the drug on Sune-1 cell (*P* < 0.05) and the cell inhibition rate of different drugs at the same concentration level had no significant difference (*P* > 0.05) (as shown in Table 1).

**Table 1:** The effect of single drug on Sune-1 cell proliferation.

Treatment	Absorbance	Cell inhibition (%)
Control	0.909 ± 0.005 a	0.0 ± 0.6 d
CDDP-L	0.659 ± 0.029 b	27.5 ± 3.5 c
CDDP-M	0.501 ± 0.013 c	44.8 ± 1.8 b
CDDP-H	0.287 ± 0.001 d	68.4 ± 1.1 a
Met-L	0.663 ± 0.022 b	27.0 ± 2.8 c
Met-M	0.478 ± 0.056 c	47.4 ± 6.2 b
Met-H	0.282 ± 0.020 d	69.0 ± 2.2 a
Qu-L	0.642 ± 0.026 b	29.3 ± 3.2 c
Qu-M	0.465 ± 0.036 c	48.9 ± 4.3 b
Qu-H	0.299 ± 0.022 d	67.1 ± 2.8 a

### The anti-proliferation effect of two-drug combined application on Sune-1 cells

**Figure 2:**
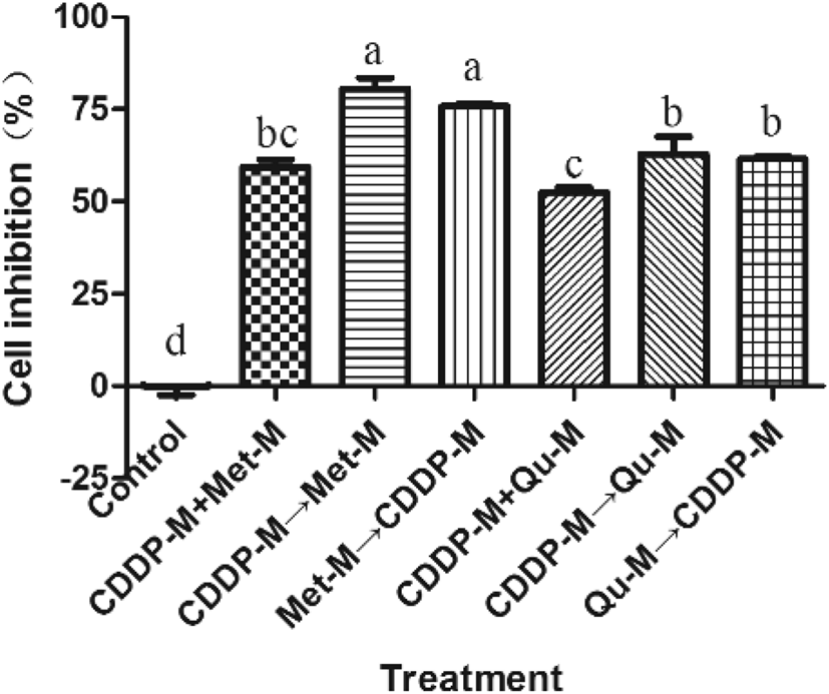
Effect of different combined application order of cisplatin and metformin, or quercetin on the proliferation of Sune-1 cells.

The anti-proliferation effect of CDDP-M and Met-M or Qu-M on Sune-1 cells under different medication sequence conditions has been determined by the CCK-8 assay. Figure 2 shows that: compared with the control group, the combination of the two drugs has a significant inhibitory effect on Sune-1 cells (*P* < 0.01). The inhibitory effect of the combined application of CDDP-M and Met-M is significantly better than that of the combined application of CDDP-M and Qu-M (*P* < 0.05), of which the sequential combination of CDDP-M → Met-M is the highest (As shown in Fig. 2). Therefore, we initially chose the CDDP → Met sequential combination method for follow-up research.

The effect of sequential application of different concentrations of cisplatin and metformin on Sune-1 cells was further determined. The results show that the sequential combination of different concentrations of cisplatin and metformin all have significant inhibitory effects on Sune-1 cells (*P* < 0.01), which is significantly better than that of single drug on Sune-1 cells (*P* < 0.05), where the sequential combination of CDDP-M and Met-H has the greatest inhibitory effect on Sune-1 cells. In terms of the combined effect of the two drugs, the sequential combination of CDDP-L and Met-M shows a good synergistic effect on the inhibition of Sune-1, and the combined application of the other groups all show antagonism (as shown in Table 2).

**Table 2:** Effects of different concentrations of cisplatin and metformin on the proliferation of Sune-1 cells.

Treatment	Absorbance	Cell inhibition (%)	fp
Control	0.855 ± 0.062		
CDDP-L → Met-L	0.532 ± 0.006	38.2 ± 0.7 g	0.81 ± 0.07 Δ
CDDP-L → Met-M	0.304 ± 0.008	64.6 ± 0.9 b	1.06 ± 0.13 *
CDDP-L → Met-H	0.422 ± 0.008	50.9 ± 0.9 de	0.66 ± 0.03 Δ
CDDP-M → Met-L	0.407 ± 0.002	52.7 ± 0.2 d	0.88 ± 0.02 Δ
CDDP-M → Met-M	0.445 ± 0.006	48.3 ± 0.7 e	0.68 ± 0.04 Δ
CDDP-M → Met-H	0.239 ± 0.006	72.2 ± 0.7 a	0.87 ± 0.03 Δ
CDDP-H → Met-L	0.475 ± 0.021	43.6 ± 2.5 f.	0.57 ± 0.04 Δ
CDDP-H → Met-M	0.353 ± 0.013	57.8 ± 1.5 c	0.69 ± 0.05 Δ
CDDP-H → Met-H	0.361 ± 0.034	56.8 ± 4.0 c	0.63 ± 0.06 Δ

Δ indicates that the combined effect of the two drugs is antagonistic; * indicates that the combined effect of the two drugs is synergistic.

### Anti-proliferation effect of three-drug combined application on Sune-1 cells

**Figure 3:**
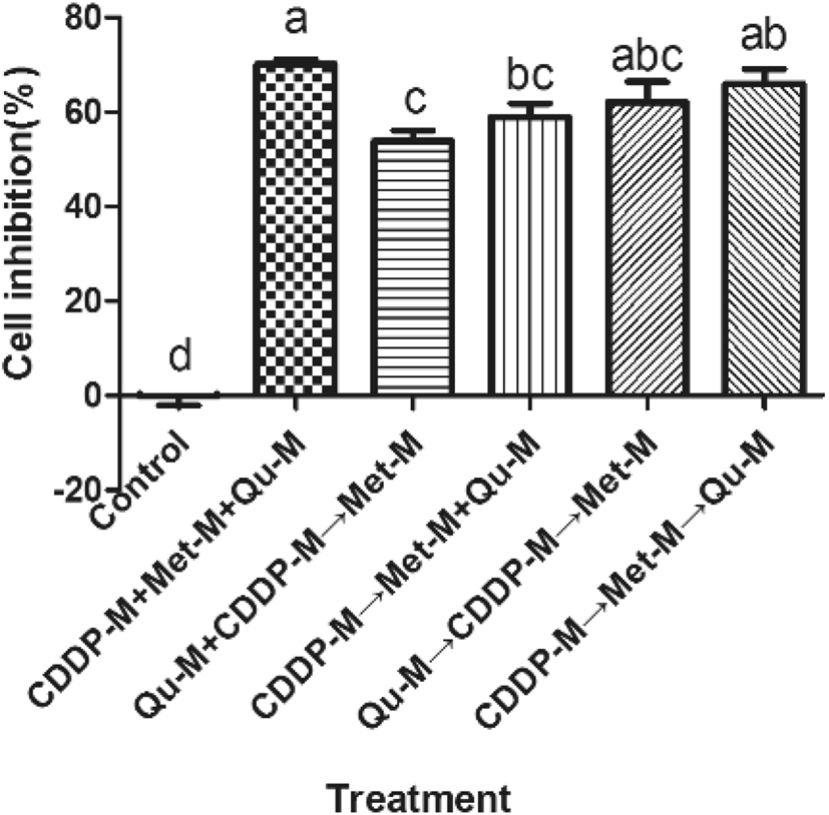
Effect of different combined application order of cisplatin, metformin, and quercetin on the proliferation of Sune-1 cells.

The CCK-8 experiment was used to determine the effects of CDDP-M, Met-M and Qu-M simultaneous and sequential combined application on the proliferation of Sune-1 cells. The results show that: CDDP-M, Met-M, and Qu-M combined application regimens all have significant inhibitory effects on Sune-1 cells (*P* < 0.01); Moreover, the anti-proliferation effect of the simultaneous combined application of the three drugs on Sune-1 cells significantly better than the sequential combination (*P* < 0.05) (as shown in Fig. 3). Therefore, we preliminarily chose the dosing method of simultaneous application of cisplatin, metformin and quercetin for subsequent research.

The L16(4^3^) orthogonal experiment was used to determine the optimal concentration level of the combination of these three drugs and the contribution level of each drug action. The results show that the combination of cisplatin, metformin, and quercetin at different concentration levels has a significant inhibitory effect on Sune-1 cells, and it is significantly better than the single drug and the combination of two drugs on the cell. Among them, the combination of CDDP-H, Met-M, and Qu-L has the greatest inhibitory effect on the proliferation of Sune-1 cells (79.1%) (as shown in Table 3). Further, by calculating the range of the test index under various factors and levels, the results show that the range corresponding to cisplatin is the largest and quercetin is the smallest. It shows that when these three drugs are used together, the contribution of cisplatin is the largest, and quercetin is the smallest (as shown in Table 4).

**Table 3:** L16 (4^3^) orthogonal scheme and results of cisplatin, metformin and quercetin combination.

Treatment	CDDP	Met	Qu	Cell inhibition (%)
1	1	1	1	3.6 ± 5.3 h
2	1	2	2	23.3 ± 11.9 g
3	1	3	3	31.5 ± 7.3 g
4	1	4	4	47.7 ± 1.0 ef
5	2	1	2	43.5 ± 2.8 f.
6	2	2	1	47.8 ± 10.0 ef
7	2	3	4	61.85 ± 4.3 cd
8	2	4	3	51.9 ± 11.4 def
9	3	1	3	55.0 ± 1.9 de
10	3	2	4	55.0 ± 5.9 de
11	3	3	1	67.5 ± 2.8 bc
12	3	4	2	67.4 ± 8.3 bc
13	4	1	4	69.6 ± 4.7 abc
14	4	2	3	69.3 ± 2.9 abc
15	4	3	2	79.1 ± 0.6 a
16	4	4	1	73.4 ± 3.6 ab

The 4 levels of each drug are as follows: CDDP 1 = 0.0, CDDP 2 = 3.3, CDDP 3 = 7.6 and CDDP 4 = 8.6 (μg/mL). Met 1 = 0.0, Met 2 = 3.4, Met 3 = 7.7 and Met 4 = 8.7 (mmol/L). Qu 1 = 0.0, Qu 2 = 53, Qu 3 = 63 and Qu 4 = 73 (μmol/L).

**Table 4:** Range analysis of L16 (4^3^) orthogonal test.

Level	K	k	R
CDDP 1	105.9	26.5	46.2
CDDP 2	205.1	51.3	
CDDP 3	244.9	61.2	
CDDP 4	291.4	72.9	
Met 1	171.7	42.9	17.2
Met 2	195.3	48.8	
Met 3	240.0	60.0	
Met 4	240.4	60.1	
Qu 1	192.3	48.1	9.9
Qu 2	152.5	38.1	
Qu 3	153.0	38.3	
Qu 4	171.9	43.0	

K represents the data sum of the test index under each factor and level; k represents the average value of the test index under each factor and level; R represents the extreme value.

### Inhibitory effect of different combined regimens of cisplatin, metformin, and quercetin on xenograft tumors

**Figure 4:**
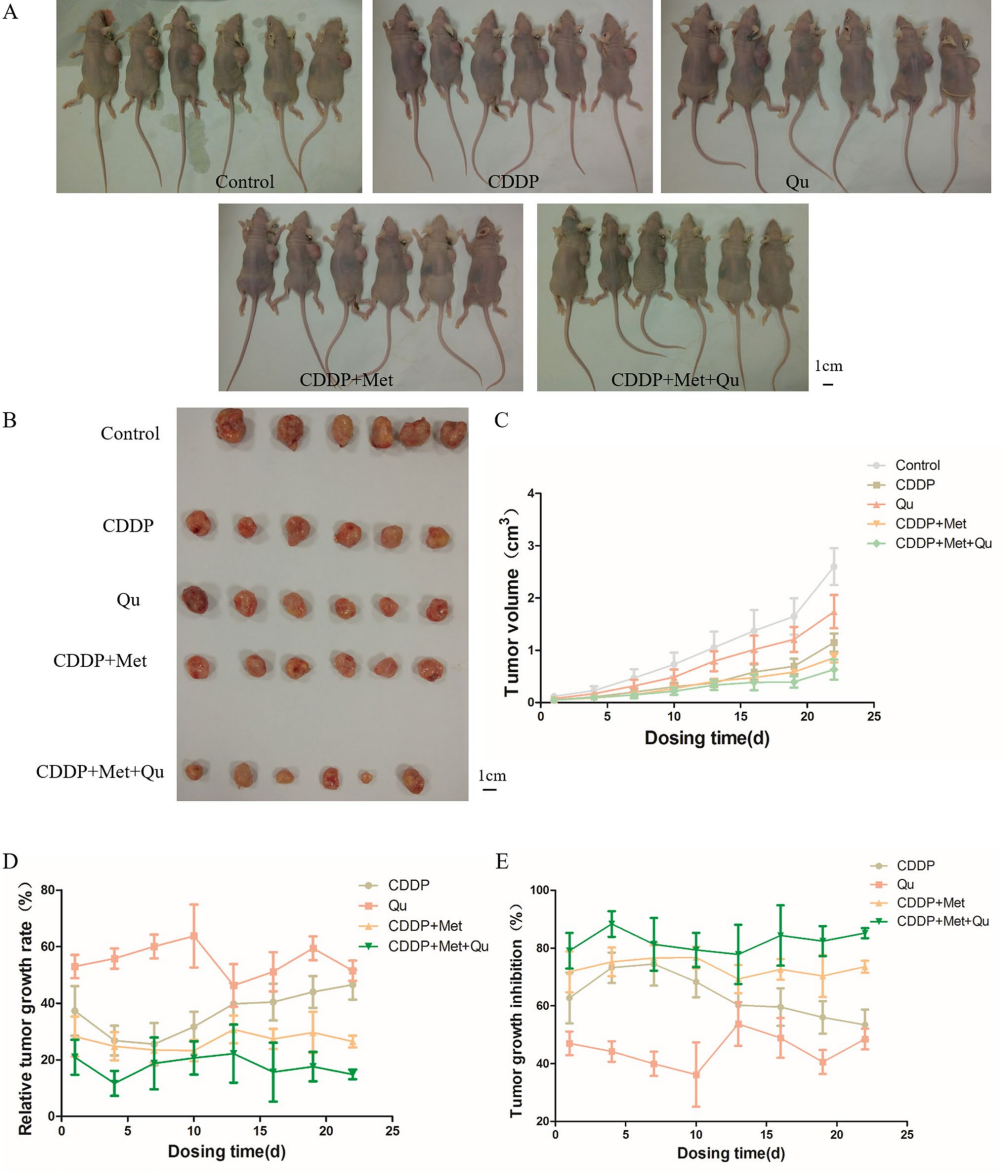
The effect of different combined regimens of cisplatin, metformin, and quercetin on the growth of xenograft tumor. (**A**, **B**) Growth of xenograft tumors in nude mouse after 8 times of dosing, (**C**) Tumor volume at different dosing time, (**D**) relative tumor growth rate at different dosing times, (**E)** tumor growth inhibition at different dosing time.

At the animal level, through the establishment of a subcutaneous xenograft nude mouse model and different drug treatments, the differences in tumor volume, relative tumor growth rate and relative tumor growth inhibition rate of nude mouse with different drug dosing regimens have been analyzed. The results show that there is no significant difference in tumor size between nude mice before injection of the drugs (*P* > 0.05), suggesting that the tumors in each group were comparable. After the treatment, the relative tumor volumes of the CDDP group, Qu group, CDDP + Met group, and CDDP + Met + Qu group are 1.7, 2.2, 1.4, and 1.2 cm^3^, respectively, compared with the control group (2.5 cm^3^) There is a significant difference (*P* < 0.05), and the tumor volume of the CDDP + Met + Qu group is significantly lower than that of other experimental groups (as shown in Fig. 4A–D). From the analysis of the relative growth inhibition rate of tumors, compared with the control group, the drug-treatment groups all show significant anti-tumor effects. The tumor growth inhibition rate of each drug-treatment group from high to low was CDDP + Met + Qu group > CDDP + Met group > CDDP group > Qu group (as shown in Fig. 4E). It can be seen that the combination of cisplatin, metformin, and quercetin is an effective combination of chemotherapy.

## Discussion

The 5-year overall survival rate of patients with nasopharyngeal carcinoma is still only about 65%. At the same time, the incidence of local recurrence and distant metastasis is still high, 19% and 20%, respectively^21^. Therefore, it is necessary to find new medications to improve the survival rate of patients with nasopharyngeal carcinoma. Many studies have shown that combination therapies composed of anticancer drugs and drugs with different mechanisms of action can produce synergistic effects and are generally more effective than monotherapy^22,23^. In this experiment, we observed that the combination of cisplatin and metformin, or quercetin has a good inhibitory effect on Sune-1 cells, and that cisplatin and metformin have a good synergistic effect under certain concentration conditions, which may be The different mechanisms of action of the two drugs are related.

Cisplatin is a complex of transition metal elements with a planar square structure, which has the characteristics of the broad anticancer spectrum, strong effect, and synergistic effect with various antitumor drugs^24^. Due to the low concentration of Cl^-^ in the cytoplasm, after cisplatin enters tumor cells, first two Cl^−^ are replaced by the OH^−^, and then OH^-^ is replaced by the nitrogen-containing base in the DNA molecular chain. Cisplatin and cancer cell DNA molecules The N7 atom of guanine and adenine inside complexes to form intra-chain compounds or inter-chain compounds, thereby preventing the replication of cancer cell DNA^25^. In addition, some studies have shown that mitochondrial DNA may be one of the important pharmacological targets of cisplatin. Due to the lack of histone mitochondrial DNA and cisplatin to form a complex, the mitochondria cannot complete nucleotide excision repair, resulting in the death of cancer cells^26^. In addition, some studies have shown that there are multiple targets for the inhibition of cisplatin on nasopharyngeal carcinoma cells, such as: KRAS^27^, Rsf-1^28^, BEX3^29^, PTEN^30^, etc.

Metformin is a classic anti-diabetic drug, which has been increasingly found to have inhibitory or preventive effects on tumors in recent years^31^. Studies have shown that metformin can reduce the resistance of nasopharyngeal cancer cells by inhibiting the DNA damage repair pathway and further inhibit its growth^32^. Secondly, metformin can directly activate adenosine monophosphatase-activated protein kinase pathway by reducing insulin-like growth factor levels, inhibit insulin signaling, thereby reducing tumor glucose supply and playing a role in inhibiting tumor growth^33^. Another anti-tumor mechanism of metformin is that it can inhibit the expression of MALAT1 and induce the expression of miR-142-3P, destroying the MALAT1/miR-142-3p cavernous body in the nucleus of cancer cells, resulting in the release of miR-142-3p into the cytoplasm, Combined with HMGA2 3′UTR, finally inhibited the invasion and migration of cancer cells^34^. In addition, in studies related to nasopharyngeal carcinoma, it was found that metformin can act on multiple cellular targets to achieve the effect of inhibiting nasopharyngeal carcinoma cells, such as AMPK^35^, E-cadherin^36^, MMP-9^36^, PECAM-1^37^, etc. This shows that metformin has a synergistic effect on chemotherapy for cancer.

Quercetin is a flavonoid compound widely present in plants and food. In recent years, its chemopreventive and therapeutic effects on cancer have gradually been recognized and attracted widespread attention. Studies have shown that quercetin has both anti-carcinogenic, anti-cancer and differentiation-inducing effects^38^. By changing the gene expression and activity of metabolic enzymes such as cytochrome p4501A1, quercetin can inhibit the activation of carcinogens, or by inhibiting the level of mRNA and protein of cyclooxygenase-2, thereby reducing cell damage and cancer action, which has Preventive effect^39,40^. In addition, in the treatment of cancer, quercetin can induce tumor cell apoptosis, regulate tumor cell cycle, interfere with tumor cell signaling pathways, act on estrogen receptors, inhibit tumor angiogenesis, inhibit tumor growth and metastasis, Enhance the sensitivity of anti-cancer drugs to tumor cells and reverse the resistance of anti-cancer drugs^41^. In addition, studies have shown that quercetin can also interfere with the occurrence and development of nasopharyngeal carcinoma through multiple cellular targets, such as EGF^42^, NF-κb^42^, HSP70/HSP90^43^, AMPK^44^, etc. In summary, quercetin has a significant synergistic effect in cancer chemotherapy.

In order to study the optimal combination of cisplatin and metformin, we designed a sequential combination of different orders and different doses. We found that the sequential combination of cisplatin and metformin in small doses showed good synergy, indicating that small doses Metformin has a certain sensitizing effect on cisplatin, which suggests that in the clinical application of the two drugs, the combination of higher doses is not better. This low-dose sequential chemotherapy regimen is beneficial under the premise of ensuring the efficacy. It can alleviate toxic and side effects, have a better tolerance, and provide an experimental basis for clinical application. However, whether the sequential application of cisplatin to metformin combined chemotherapy can achieve better benefits for patients with nasopharyngeal carcinoma and the optimal sequential combined dose is worthy of further study. The synergistic mechanism of this sequential combination is still unclear. To be further studied.

In this experiment, the combination of cisplatin, metformin, and quercetin showed strong antitumor activity, which is significantly better than the combination of two drugs and single drug, suggesting that the combination of three drugs will become a highly effective chemotherapy for nasopharyngeal carcinoma regimen. A number of studies have shown that the combination of three drugs with significant synergistic effects will have a significant improvement in the efficacy of single drugs or second drugs. For example: Huang Lin et al., have found that the use of cisplatin, paclitaxel, and fluorouracil in the treatment of distant metastatic nasopharyngeal carcinoma is more effective and has a higher tolerance for adverse reactions^45^. Ge et al., have found that in clinical studies, cisplatin the combination of epirubicin and fluorouracil for the treatment of nasopharyngeal carcinoma has a higher efficacy, and its main toxic side effect-hematological toxicity WBC is significantly reduced, and the remaining toxic side effects are not serious^46^. This suggests that the combined application of cisplatin, metformin, and quercetin is a potential chemotherapy for the treatment of nasopharyngeal carcinoma, and the combined application of the three can complement each other to enhance the anti-tumor effect. This experiment shows that the contribution levels of the three drugs in the combined application of the three drugs are cisplatin, metformin, and quercetin from high to low, which provides a theoretical basis for the optimal dose distribution of the three drugs in the subsequent clinical research.

## Data Availability

No additional data are available.
